# Elevated Plasma Soluble PD-L1 Levels in Out-of-Hospital Cardiac Arrest Patients

**DOI:** 10.3390/jcm10184188

**Published:** 2021-09-16

**Authors:** Miho Sumiyoshi, Eiji Kawamoto, Yuki Nakamori, Ryo Esumi, Kaoru Ikejiri, Toru Shinkai, Yuichi Akama, Asami Ito-Masui, Hiroshi Imai, Arong Gaowa, Eun Jeong Park, Motomu Shimaoka

**Affiliations:** 1 Department of Anesthesia, Mie Prefectural General Medical Center, 5450-132 Hinaga, Yokkaichi 510-0885, Mie, Japan; sumikichimiho@gmail.com; 2 Department of Molecular Pathobiology and Cell Adhesion Biology, Mie University Graduate School of Medicine, 2-174 Edobashi, Tsu-City 514-8507, Mie, Japan; yunakamori@gmail.com (Y.N.); ryoo1582@hotmail.co.jp (R.E.); y-akama@nms.ac.jp (Y.A.); shironeko1am@yahoo.co.jp (A.I.-M.); arong-g@doc.medic.mie-u.ac.jp (A.G.); epark@med.mie-u.ac.jp (E.J.P.); 3 Department of Disaster and Emergency Medicine, Mie University Graduate School of Medicine, 2-174 Edobashi, Tsu-City 514-8507, Mie, Japan; kaoru.ikejiri@gmail.com (K.I.); trs2006@hotmail.co.jp (T.S.); 3999101@gmail.com (H.I.)

**Keywords:** post-cardiac arrest syndrome, immunosuppression, inflammation, PD-1, PD-L1, lymphocytes

## Abstract

**Background:** A deregulated immune system has been implicated in the pathogenesis of post-cardiac arrest syndrome (PCAS). A soluble form of programmed cell death-1 (PD-1) ligand (sPD-L1) has been found at increased levels in cancer and sustained inflammation, thereby deregulating immune functions. Here, we aim to study the possible involvement of sPD-L1 in PCAS. **Methods:** Thirty out-of-hospital cardiac arrest (OHCA) patients consecutively admitted to the ER of Mie University Hospital were prospectively enrolled. Plasma concentrations of sPD-L1 were measured by an enzyme-linked immunosorbent assay in blood samples of all 30 OHCA patients obtained during cardiopulmonary resuscitation (CPR). In 13 patients who achieved return-of-spontaneous-circulation (ROSC), sPD-L1 levels were also measured daily in the ICU. **Results:** The plasma concentrations of sPD-L1 in OHCA were significantly increased; in fact, to levels as high as those observed in sepsis. sPD-L1 levels during CPR correlated with reduced peripheral lymphocyte counts and increased C-reactive protein levels. Of 13 ROSC patients, 7 cases survived in the ICU for more than 4 days. A longitudinal analysis of sPD-L1 levels in the 7 ROSC cases revealed that sPD-L1 levels occurred in parallel with organ failure. **Conclusions:** This study suggests that ischemia- reperfusion during CPR may aberrantly activate immune and endothelial cells to release sPD-L1 into circulation, which may play a role in the pathogenesis of immune exhaustion and organ failures associated with PCAS.

## 1. Introduction

Post-cardiac arrest syndrome (PCAS), which occurs in resuscitated patients undergoing cardiac arrest, is characterized by four key pathological manifestations including post-cardiac arrest brain injury, post-cardiac arrest myocardial dysfunction, systemic ischemia-reperfusion, and persistent precipitating pathophysiology [1,2,3]. Successful management of PCAS constitutes a vital component of post-cardiac arrest care, critically affecting the prognosis of resuscitated out-of-hospital cardiac arrest (OHCA) patients [4]. An ischemia-reperfusion injury that systemically occurs in PCAS induces a deregulated immune response and sustained inflammation, resembling the pathophysiology of sepsis [5]. Immune-suppression represents an important pathology involved not only in sepsis, but also in PCAS, thereby increasing the risk of such infections as pneumonia during post-cardiac arrest care [6]. Leukocytes isolated from PCAS patients have been shown to exhibit reduced TNF, IL-6, and IL-10 production [7], as well as reduced expression of leukocyte antigen DR [8]. The plasma samples of PCAS patients have been shown to contain immunosuppressive activities, thereby inhibiting cytokine production of circulating leukocytes [5]. However, the underlying immunosuppressive mechanisms associated with PCAS remain to be elucidated.

Programmed cell death-1 (PD-1) is a major check-point regulator molecule expressed on T-lymphocytes and other immune cells [9]. Upon binding of PD-1 ligand 1 (PD-L1), PD-1 transduces intracellular signaling to suppress T-lymphocyte effector functions, thereby inducing immune exhaustion [10]. PD-1/PD-L1-mediated immune exhaustion has been implicated in the pathogenesis of the types of immune evasion exhibited by cancers. This has led to the development of several cancer immune therapies aimed at inhibiting PD-1/PD-L1 interactions and reversing immune suppression [11]. PD-1/PD-L1 interactions are thought to play important roles in the immune suppression associated with sepsis, which is characterized by a deregulated immune system that induces inflammatory tissue injury, a condition exacerbated by impaired functionality in lymphoid and myeloid leukocytes [12,13]. PD-L1 exists not only on the cell surface in a membrane-bound form, but also in the plasma in a soluble form. Soluble PD-L1 (sPD-L1) retains its ability to bind to PD-1 on immune cells, thereby inducing immune exhaustion [14]. Elevated plasma levels of sPD-L1 have been reported in both cancer and inflammatory disorders [15,16,17]. One meta-analysis of cancer patients showed that higher levels of sPD-L1 are associated with poor prognoses, suggesting that plasma sPD-L1 plays a role in suppressing anti-tumor immunity [18]. We have recently documented elevated plasma levels of sPD-L1 in sepsis patients, which is associated with impaired renal, central nervous, and coagulation systems [19]. Given the potential similarities in the pathogenesis of sepsis and PCAS, we hypothesize that sPD-L1 would be elevated in OHCA patients and might correlate with the severity of PCAS. To address this, we have designed a pilot study to investigate the plasma levels of sPD-L1 in OHCA patients. 

## 2. Materials and Methods

This study protocol was reviewed and approved by the Institutional Review Board (IRB) of the Mie University Graduate School of Medicine (#3027). Informed consent to participate in this study was obtained in all cases from close family members of OHCA patients admitted to the emergency department and intensive care unit (ICU) at Mie University Hospital Japan.

### 2.1. Study Design and Patient Characteristics 

This study enrolled 30 consecutive OHCA patients from August 2020 to May 2021. Upon arrival at the hospital, all patients were immediately subjected to our institute’s standard cardiopulmonary resuscitation (CPR) protocol. Specifically, patients were intubated, manually ventilated with 100% oxygen, and treated with standard chest compressions by ICU physicians. The end-tidal carbon dioxide (EtCO2) concentrations were monitored to assure effective heart compression during CPR. Blood samples were drawn from the femoral arteries as soon as possible during CPR in all cases and from the arterial lines daily in the ICU in ROSC cases, and were used primarily for clinical purposes (e.g., laboratory testing). Parts of the blood samples were used to measure plasma cell-free PD-L1 concentrations. Platelet-free plasma fractions were prepared from blood samples as previously described [20]. Platelet free-plasma samples were kept at −80 °C until use. Patient clinical and laboratory data were extracted from electronic ICU records. In ROSC cases, the outcome from resuscitation was assessed by the cerebral performance category (CPC) score at the time of ICU exit. Whole-body, unenhanced postmortem computed tomography was performed for the cadavers of all patients to diagnose pneumonia.

### 2.2. Soluble PD-L1 Measurements 

The concentrations of sPD-L1 in the plasma samples were measured using enzyme-linked immunosorbent assay kits (Abcam, Japan, Cat#ab214565) according to the manufacturer’s instructions with known concentrations of human recombinant sPD-L1 as an internal standard [19]. Each sample was analyzed in duplicate. The plasma levels of sPD-L1 in OHCA were compared with those of healthy volunteers and sepsis patients measured by exactly the same method in our previous report [19].

### 2.3. Statistical Analyses

Statistical analyses were performed using SPSS software v.25.0 (IBM Corp, Armonk, NY, USA). The results are presented as a median ± interquartile range, unless otherwise noted. Kruskal–Wallis tests were used to compare three or more groups. Mann–Whitney tests were used for two-group comparisons. To compare the correlations, Spearman’s rank correlation was calculated between each data set. A *p*-value < 0.05 was considered statistically significant.

## 3. Results

### 3.1. Plasma PD-L1 Levels in OHCA Patients Were as High as Those in Sepsis Patients

The thirty OHCA patients enrolled in this study included 12 females and 18 males who ranged from 48 to 92 years old (75.4 ± 14.2) (Table S1). The causes of OHCA included pneumonia (8 cases), acute myocardial infarction (8 cases), thoracic aortic dissection (4 cases), chronic heart failure (4 cases), suicide by hanging (2 cases), myocarditis (1 case), subarachnoid hemorrhage (1 case), and hyperkalemia from chronic renal failure (1 case) and asphyxia (1 case) (Table 1). Twenty-six were non-shockable OHCA. Although, return of spontaneous circulation (ROSC) occurred in 13 cases, of whom 7 survived in the ICU for more than 4 days.

**Figure 1:**
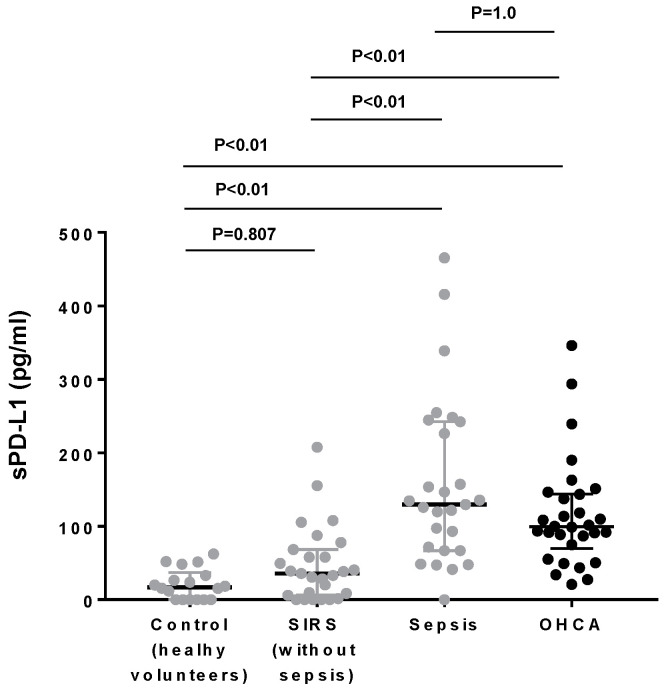
Comparisons of plasma sPD-L1 levels in OHCA, sepsis, systemic inflammatory response syndrome (SIRS), and healthy volunteers. The data in OHCA patients (black dots) were obtained in this study. For comparison, our previously reported data (gray dots) of sepsis, SIRS, and healthy volunteers measured by exactly the same method are shown.

We have studied the plasma levels of sPD-L1 as early as possible during CPR before ROSC, in which chest compressions partially restored circulation, potentially triggering ischemia-reperfusion injury [21]. We have shown that the plasma levels of sPD-L1 in OHCA during CPR were increased (Figure 1). Compared with the previous results that we reported using the exactly same methods [19], the PD-L1 concentrations in OHCA were significantly higher than those of healthy volunteers (Figure 1) and were as high as those of the sepsis patients measured by the exactly same method in our previous report (Figure 1).

**Figure 2:**
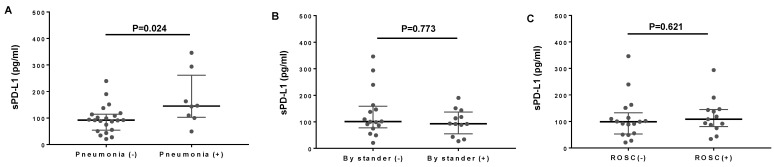
Subgroup analyses comparing plasma sPD-L1 levels in OHCA patients with or without pneumonia (**A**); OHCA patients who were treated with bystander CPR or not (**B**); and OHCA patients who subsequently underwent ROSC or not (**C**). Each black dot denotes individual patient’s data.

A sub-group analysis has shown that the presence of pneumonia was associated with increased sPD-L1 levels in OHCA during CPR (Figure 2A). Nevertheless, the sPD-L1 levels in the OHCA sub-group without pneumonia remained higher than those in healthy volunteers and SIRS without sepsis (Figure S1). Application of bystander CPR might reduce the duration of whole-body ischemia, thereby possibly mitigating reperfusion injury. However, bystander CPR did not affect sPD-L1 levels in OHCA during CPR (Figure 2B). The sPD-L1 levels in OHCA during CPR did not correlate with the subsequent occurrences of ROSC (Figure 2C).

### 3.2. Correlations of Plasma sPD-L1 Levels with Clinical Parameters in OHCA Patients

**Figure 3:**
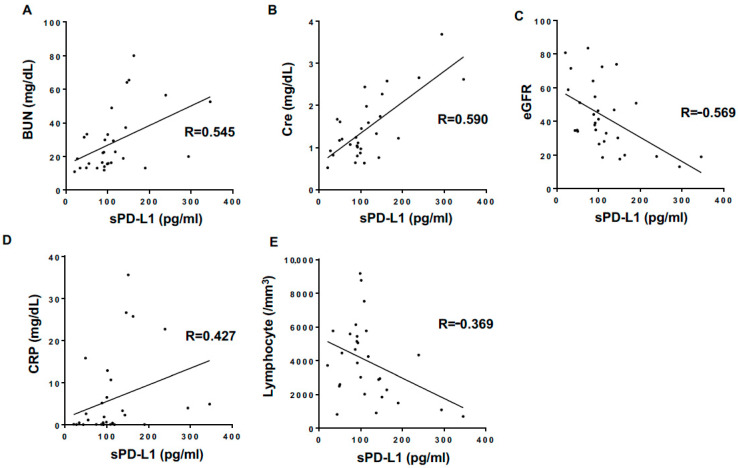
Correlations of plasma sPD-L1 levels with laboratory and clinical parameters (selected). A panel of scatter plots with a linear regression line shows statistically significant correlations of plasma sPD-L1 levels with BUN (**A**), Cre (**B**), eGFR (**C**), C-reactive protein (CRP) (**D**), and lymphocyte count (**E**).

While investigating how sPD-L1 levels affected the clinical laboratory test results sampled during CPR, we found that they correlated with increased blood urea nitrogen (BUN) and creatinine (Cre) and decreased estimated glomerular filtration rate (eGFR) values (Table 2, Figure 3). In addition, sPD-L1 levels correlated with reduced lymphocyte numbers and increased C-reactive protein levels (Table 2).

### 3.3. Longitudinal Changes of sPD-L1 Levels in ROSC Cases 

**Figure 4:**
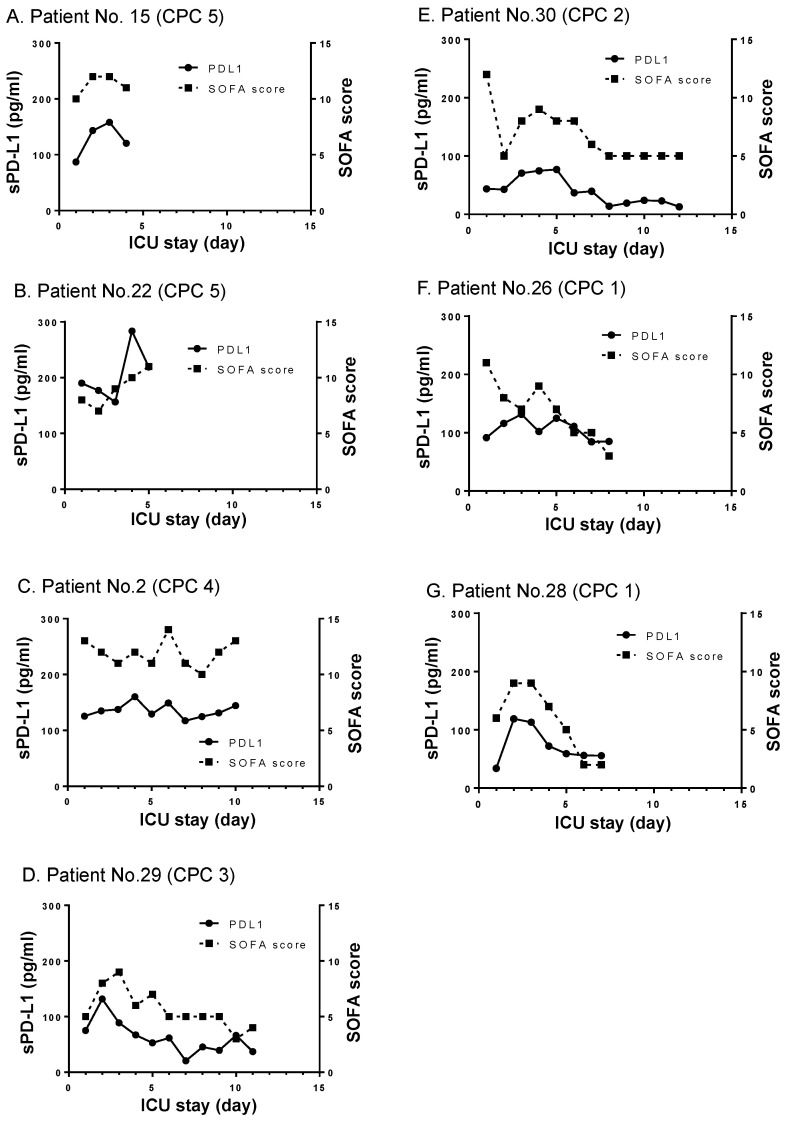
Longitudinal changes of sPD-L1 levels in ROSC cases. sPD-L1 levels (solid lines) and SOFA scores (dotted lines) are shown. The cerebral performance category (CPC) scores at the time of ICU discharge are shown. Seven ROSC cases (**A**–**G**) who stayed in the ICU for more than 4 days are presented.

To examine the longitudinal changes of sPD-L1 levels in PCAS, we studied 7 ROSC patients who survived for more than 4 days in the ICU (Figure 4) and, thereby, sought to correlate the sPD-L1 levels with the severity of organ failures and the outcome. In these cases, we found that sPD-L1 levels remained higher than normal, and appeared to correlate with the severity of organ failures as represented by the SOFA scores (Figure 4). The CPC score was used to assess the outcome of resuscitation. In the cases presenting high CPC scores [22] at the time of ICU discharge, sPD-L1 levels remained exceedingly high as did SOFA scores (Figure 4A–C). By contrast, in other cases presenting low to moderate CPC scores at the time of ICU discharge, sPD-L1 levels gradually reduced, as did SOFA scores (Figure 4E–G).

## 4. Discussion

In this pilot study involving 30 OHCA patients, we observed significantly elevated levels of plasma sPD-L1 during CPR. The sPD-L1 concentrations in OHCA were as high as those of sepsis [19], thereby supporting our hypothesis that the PD-1/PD-L1 signaling pathway might be involved in the pathogenesis of both PCAS and sepsis [12]. The increased sPD-L1 levels are thought to result from ischemia-reperfusion, because, even before ROSC, circulatory support by CPR with chest compressions partially restored tissue perfusion, thereby activating the mechanisms that lead to ischemia-reperfusion injury [21]. Infections such as pneumonia [23] and underlying medical conditions present in the current OHCA cohort (Table 1) such as diabetes mellitus [17], rheumatoid arthritis [24], and systemic lupus erythematosus [25] have been shown to associate with a modest increase in the plasma sPD-L1 levels. Elevated sPD-L1 levels were found in all OHCA cases regardless of their underlying medical conditions, thereby supporting the impact of ischemia-reperfusion injury during CPR to cause a significant increase in sPD-L1 in the plasma. However, it is possible that pneumonia and some underlying medical conditions could contribute to the increased sPD-L1 levels seen in this study. Further investigations are needed to confirm these suspicions.

sPD-L1 levels during CPR were indistinguishably elevated in both ROSC (+) and ROSC (−) patients. To further study how sPD-L1 levels unfold during PCAS, we examined 7 ROSC patients treated in the ICU for more than 4 days. In all cases, sPD-L1 levels remained high, albeit changing in proportion to the SOFA scores. This preliminary finding needs to be further substantiated in future investigations to support our contention that sPD-L1 may be included as a biomarker candidate to predict the severity of organ failures in PCAS. Of note, in sepsis patients, sPD-L1 levels have been shown to correlate with SOFA scores, as well as with specific laboratory and clinical parameters of the impaired renal, coagulation, and central nervous systems [19].

PD-L1 is expressed not only in leukocytes such as macrophages and monocytes, but also in non-leukocytic cells such as endothelial and epithelial cells [15,26]. Although the present study did not demonstrate the upregulation of activation markers for endothelial cells and leukocytes, previous studies have shown that ischemia-reperfusion injury in PCAS induces aberrant endothelial [27,28,29] and leukocyte activation [30,31]. Thus, leukocytes and endothelial cells likely release sPD-L1 in resuscitated OHCA patients. sPD-L1 is mostly produced by proteolytic cleavage of the extracellular part of membrane-bound PD-L1, although a small proportion of sPD-L1 in the plasma may originate from alternatively spliced PD-L1 mRNA lacking a transmembrane domain [15]. Several proteases such as matrix metalloproteinase (MMP)-9 [32], MMP-13 [33], a disintegrin and metalloproteinase (ADAM)10 [34], and ADAM17 [35] have been shown to cleave membrane-bound PD-L1, thereby forming sPD-L1. Notably, MMPs and ADAMs, including MMP-9 [36], MMP-13 [37], ADAM10 [38], and ADAM17 [39], have been shown to be activated in ischemia-reperfusion injury models. Thus, one plausible scenario is that the ischemia-reperfusion injury that occurs during CPR activates MMPs and ADAMs, which cleave off PD-L1 on leukocytes and endothelial cells to produce sPD-L1.

As sPD-L1 retains the ability to bind PD-1 on the cell surface, thereby transmitting signals to dampen immune cell activation [15], increased levels of sPD-L1 have been implicated in the perturbed anti-tumor immunity observed in cancer patients [11] and in the immune paralysis suffered by sepsis patients [19]. Increased sPD-L1 in OHCA patients may constitute an important component of the immunosuppressive milieu in the plasma, as previously suggested in cases of PCAS [5]. In this way, aberrant activation of PD-1 signaling by the increased expression of sPD-L1 could give rise to unwanted immune suppression, which could predispose patients to opportunistic infections [12]. Activation of PD-1 signaling by PD-L1 induces not only T cell unresponsiveness, but also apoptosis of CD4 [40] and CD8 [41] T cell subsets. PD-1 is also expressed in B cells [42] and activation-induced apoptosis of memory B cells has been reported in critically ill patients [43]. Thus, studying the potential depletion of different lymphocyte subsets in OHCA would be of great interest.

Alternatively, increased levels of sPD-L1 may have regulatory and protective properties, as the PD-1 signaling elicited by PD-L1 alleviates ischemia-reperfusion injuries to the kidney [44] and liver [45]. However, conflicting results have been reported in ischemia-reperfusion in the brain. PD-1 deficiencies in knockout mice worsened brain infarction in cases of ischemia-reperfusion, supporting the protective role played by PD-1 signaling [46]. By contrast, PD-L1 deficiencies in knockout mice reduced brain infarction [47]. Thus, the roles of PD-1 signaling, as activated by PD-L1 during ischemia-reperfusion, may vary depending on the types of organs. This could partly be explained by the deregulated balance between effector and regulatory T cells in PCAS [48], as PD-1 signaling induces opposing effects in each [49]. Whereas PD-1 signaling in effector T-cell function suppresses inflammation, the signaling in regulatory T-cells results in the inhibition of immune-suppressive effects, thereby augmenting inflammation [49].

A potential limitation of the present study is the concern that age differences could explain the cause of the increased sPD-L1 levels in the OHCA cohort, as the OHCA cohort is older than the healthy volunteer cohort (Table S1). The previous study that measured plasma sPD-L1 concentrations in healthy volunteers of different age groups reported that the concentrations between the 31–50-year-old group and that of the 51–70-year-old group were similar [50], thereby suggesting a modest effect, if any, of ages in the present results. However, as age-related underlying medical conditions could also affect the sPD-L1 measurements, further investigations involving an increased number of OHCA patients and age-matched cases with similar underlying medical conditions are needed to address the concern. Another potential limitation is that one should be cautious in interpreting the correlations of sPD-L1 with certain clinical laboratory test results (e.g., increased BUN, increased Cre, and decreased lymphocyte counts) and increased C-reactive protein levels in blood samples obtained during CPR, as such test results may simply reflect the severity of pre-existing diseases and/or the severity of ischemia-reperfusion.

## 5. Conclusions

In summary, this study supports our hypothesis that elevated levels of sPD-L1 in OHCA patients during CPR reflect systemic ischemia-reperfusion, which aberrantly induces immune and endothelial cells to shed the extracellular part of PD-L1 into the circulation. Our results suggest that sPD-L1 may play a role in the pathogenesis of immune exhaustion and the multi-organ failures associated with PCAS. Thus, sPD-L1 could be added to the list of reported biomarkers of PCAS [21,28,30,51,52,53,54,55]. Follow-up control studies involving a large cohort of OHCA patients are needed to validate our hypothesis.

## Figures and Tables

**Table 1:** Demographic characteristics of OHCA patients.

Patient	Cause of OHCA	Age	Gender	UMC	sPDL1 (pg/mL)	EMS ^1^ (mins)	Hospital ^2^ (mins)	Bystander CPR	Rhythms ^3^	ROSC ^4^	CPC Scale
1	AD	87	F	HT	101.5	9	27		N		
2	Myocarditis	48	M	HL	137.5	6	18		N	+ ^4^	4
3	Pneumonia	80	M	DM	146.4	8	39		N	+	5
4	SAH	63	F		108.5	12	25	+	N	+	
5	CHF	70	M		50.3	11	27	+	S		
6	Pneumonia	89	M		109.7	12	38		N		
7	AD	90	F	HT	92.1	9	15	+	N		
8	CHF	82	M	OMI	88.6	12	30	+	N		
9	AMI	92	F	DM	93.4	11	30	+	N	+	5
10	Hanging	87	F	RA	98.9	7	20		N		
11	AD	90	F	AF, TAA	91.0	8	31		N		
12	AD	62	M	DM, CKD	113.6	9	22	+	N		
13	Hyperkalemia	65	F	SLE, CKD, HT	151.3	12	23	+	N		
14	Pneumonia	75	F	CI, OMI	162.8	9	31		N		
15	AMI	91	F	DM, HT	87.0	17	29		N	+ ^4^	5
16	Pneumonia	85	M	HT	346.0	6	19		N		
17	AMI	83	M	HT, OMI	118.3	9	26	+	N	+	5
18	Pneumonia	82	M	DM	143.3	5	20	+	N	+	5
19	CHF	78	M		239.3	7	29		N		
20	AMI	89	F		20.7	8	14		N		
21	Pneumonia	76	F		49.1	17	41		N		
22	Hanging	50	M	Depression	190.1	6	30	+	N	+ ^4^	5
23	Pneumonia	86	F		100.1	11	29		N		
24	Asphyxia	88	M		27.4	11	21	+	N		
25	Pneumonia	86	M	DM, HT, CKD	293.8	9	27		N	+	5
26	AMI	52	M	DM, HT, AF	91.6	10	28	+	S	+ ^4^	1
27	AMI	52	M		55.3	6	21		N		
28	AMI	69	M	CI	33.9	6	29	+	S	+ ^4^	1
29	AMI	58	M	CHF	74.8	8	15		S	+ ^4^	3
30	CHF	58	M	HL, OMI, AF	43.6	11	28	+	N	+ ^4^	2

OHCA: out-of-hospital cardiac arrest, sPD-L1: soluble PD-L1, EMS: emergency medical services, CPR: cardio pulmonary resuscitation, ROSC: return of spontaneous circulation, ICU: intensive care unit. AD: aortic dissection, SAH: subarachnoid hemorrhage, CHF: congestive heart failure, AMI: acute myocardial infarction, UMC: underlying medical conditions, CPC scale: cerebral performance category scale, HT: hypertension, HL: hyperlipidemia, DM: diabetes mellitus, OMI: old myocardial infarction, RA: rheumatoid arthritis, AF: atrial fibrillation, TAA: thoracic aortic aneurysm, CKD: chronic kidney disease, SLE: systemic lupus erythematosus, CI: cerebral infarction. All patients were transported to emergency department with ongoing CPR. ^1^ EMS arrival time (defined as the interval from 1-1-9 call receipt to EMS arrival); ^2^ time from EMS arrival at scene to EMS arrival at hospital; ^3^ N, non-shockable; S, shockable; ^4^ stayed in the ICU for more than 4 days.

**Table 2:** Correlations of soluble PD-L1 with laboratory and clinical parameters in OHCA patients.

	^1^ R Values Correlated with sPD-L1
Total protein	−0.147
Albumin	−0.199
Blood urea nitrogen	0.545 *
Creatinine	0.590 *
Estimated glomerular filtration rate	−0.569 *
Na	0.190
K	0.172
Cl	0.141
Aspartate transaminase	0.186
Alanine transaminase	0.062
Total bilirubin	0.147
C-reactive protein	0.427 *
White blood cell counts	−0.236
Red blood cell counts	−0.221
Haemoglobin	−0.101
Haematocrit	−0.123
Neutrophil	−0.166
Lymphocyte	−0.369 *
Monocyte	−0.258
Platelet	0.137
Activated partial thromboplastin time	−0.102
Prothrombin time	−0.015
Prothrombin time (%)	0.012
Prothrombin time-international normalized ratio	−0.022
Fibrinogen	0.098
D-dimer	0.058
pH	0.049
Partial pressure of arterial oxygen	0.171
Partial pressure of arterial carbon dioxide	−0.152
HCO_3_^−^	0.015
Lactate	0.156
Troponin I	0.069
^2^ EMS arrival time	−0.278
^3^ EMS-to-hospital time	0.072

^1^ Levels of correlations (i.e., R values) are shown for each pair. Values in bold indicate significant difference (* *p* < 0.05). ^2^ EMS arrival time (defined as the interval from 1-1-9 call receipt to EMS arrival). ^3^ Time from EMS arrival at scene to EMS arrival at hospital.

## Data Availability

The data presented in this study are available on request from the corresponding author.

## Supplementary Materials

The following are available online at https://www.mdpi.com/article/10.3390/jcm10184188/s1, Figure S1. Comparisons of plasma sPD-L1 levels in the subgroup of OHCA without pneumonia, with sepsis, systemic inflammatory response syndrome (SIRS), and healthy volunteers; Table S1. Clinical parameters of study participants.
