# Supplementary material for: Elevated Plasma Soluble PD-L1 Levels in Out-of-Hospital Cardiac Arrest Patients
Source: J Clin Med. 2021 Sep 16;10(18):4188. doi: 10.3390/jcm10184188 (PMC8468744; doi:10.3390/jcm10184188)
Supplement: Supplementary file 1 [file jcm-10-04188-s001.zip › jcm-1353039Fig S1.pdf]

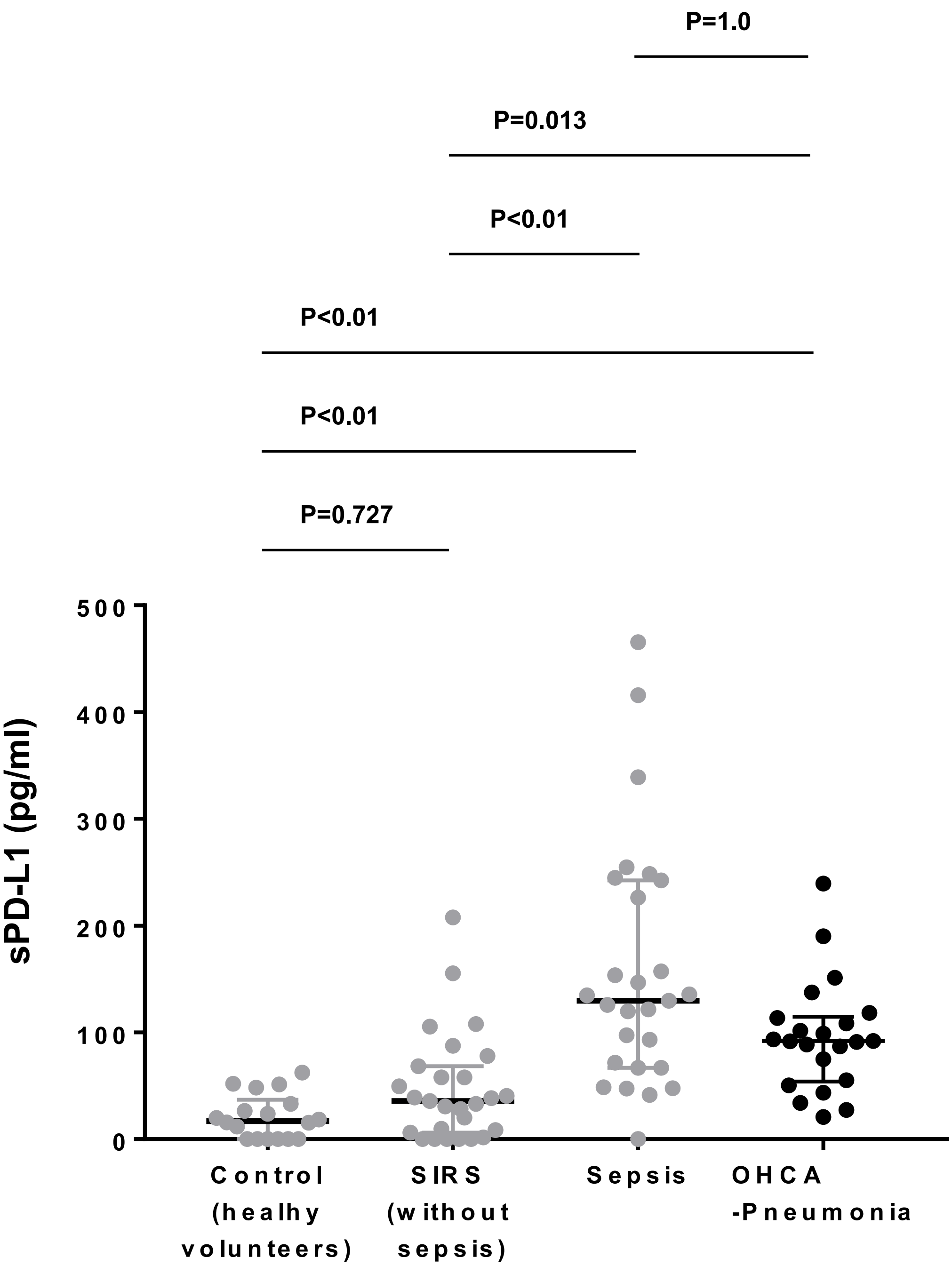

Figure S1. Comparisons of plasma sPD-L1 levels in the subgroup of OHCA without pneumonia, with sepsis, systemic inflammatory response syndrome (SIRS), and healthy volunteers. The data in the OHCA patients (black dots) were obtained in this study. For comparison, our previously reported data (gray dots) of sepsis, SIRS, and healthy volunteers measured by exactly the same method are shown.
