# Supplementary material for: Elevated Plasma Soluble PD-L1 Levels in Out-of-Hospital Cardiac Arrest Patients
Source: J Clin Med. 2021 Sep 16;10(18):4188. doi: 10.3390/jcm10184188 (PMC8468744; doi:10.3390/jcm10184188)
Supplement: Supplementary file 1 [file jcm-10-04188-s001.zip › jcm-1353039Table S1.pdf]

Table S1. Clinical parameters of study participants

	Control(healthy volunteers), N=18	SIRS(without sepsis), N=27	Sepsis, N=27	OHCA, N=30	P value
**Age, years**	49.8±14.5	57.7±20.6	68. 4 ±18.2	75.4±14.2	<0.001
**Gender Male/Female**	8/10	10/17	11/16	18/12	0.859
**sPDL1 (pg/ml)**	21.0±4.8	46.9±9.8	157.1±4.2	115.3±13.5	<0.001
**TP, g/dL**		6.4±0.2	5.5±0.2	6.1±0.2	0.012
**Alb, g/dL**		3.5±0.1	2.7±0.1	3.0±0.1	<0.001
**BUN, mg/dL**		15.4±1.4	27.7±3.2	28.6±3.4	<0.001
**Cre, mg/dL**		0.8±0.1	1.1±0.1	1.5±0.1	<0.001
**AST, U/L**		50.8±10.4	137.9±47.2	716.6±182.2	<0.001
**ALT, U/L**		42.0±9.6	98.1±36.7	402.9±90.6	<0.001
**T-bil, mg/dL**		1.0±0.1	1.8±0.8	0.82±0.1	0.531
**CRP, mg/dL**		5.6±1.4	12.4±1.6	6.2±1.8	0.005
**WBC count, ×10^9/L**		11.4±0.9	14.8±1.2	11.1±0.9	0.335
**Hb, g/dL**		11.8±0.48	10.7±0.3	11.3±0.5	0.204
**Hct, %**		35.3±1.3	32.3±1.0	38.9±1.6	0.031
**Neutrophil, ×10^9/L**		9.0±0.9	12.8±8.1	6.3±0.7	0.002
**Lymphocyte, ×10^9/L**		1.6±0.2	0.5±1.5	4.0±0.4	<0.001
**Monocyte, ×10^9/L**		0.6±0.2	0.5±0.1	0.5±0.1	0.519
**Platelet, ×10^3/μL**		215.6±19.3	175.4±22.9	176.6±16.9	<0.001
**SOFA score**		5.5±0.7	8.4±0.9	-	0.016

Results are expressed as mean +/- SEM. The following abbreviations are used. TP: total protein, Alb: albumin, SOFA score: sequential organ failure assessment score.
